# Co‐Manipulation of Ultrafine Nanostructure and Uniform Carbon Layer Activates Maricite‐Structured NaFePO_4_ as a High‐Performance Cathode for Sodium‐Ion Batteries

**DOI:** 10.1002/smsc.202300122

**Published:** 2023-11-20

**Authors:** Liping Zhao, Lai Yu, Guanglin Wan, Nazir Ahmad, Xinyi Ma, Zongzhi Tao, Genqiang Zhang

**Affiliations:** ^1^ Hefei National Research Center for Physical Sciences at the Microscale CAS Key Laboratory of Materials for Energy Conversion Department of Materials Science and Engineering University of Science and Technology of China Hefei Anhui 230026 China

**Keywords:** carbon coatings, cathodes, maricite NaFePO_4_, nanoparticles, sodium-ion batteries

## Abstract

Sodium iron phosphate (NaFePO_4_) has attracted significant attention because of its high theoretical capacity (155 mA h g^−1^), remarkable structural stability, and abundant elemental composition. However, the electrochemical reversibility of maricite NaFePO_4_ is generally considered inactive. Herein, a nanoengineering strategy to activate the electrochemical activity of maricite NaFePO_4_ is presented. This approach involves the construction of ultrasmall maricite NaFePO_4_ nanoparticles encapsulated within an ultrathin carbon layer (denoted as m‐NFP@C), which greatly improves the electrochemical properties of the material. Notably, the optimized m‐NFP@C nanoparticles exhibit an impressive reversible capacity of 101.4 mA h g^−1^ after 100 cycles at a current density of 20 mA g^−1^, demonstrating a remarkable capacity retention of 90.5%. Furthermore, when coupled with the bismuth–carbon microfoam‐like compound (Bi@NC‐MF) anode, the fabricated sodium‐ion full cell exhibits exceptional cycling stability with a capacity retention of 90.6% over 250 cycles. The remarkable electrochemical performance of this material can be attributed to its excellent structural stability, ultrafine nanostructure, and uniform carbon coating, which effectively shorten the Na^+^ diffusion pathways, prevent the aggregation and fragmentation of nanoparticles, and enhance electronic conductivity. This work is anticipated to open up a new route for activating maricite NaFePO_4_ and advancing the development of polyanion‐type electrode materials.

## Graphical abstract

Carbon‐coated maricite NaFePO_4_ nanoparticles (denoted as *m*‐NFP@C) are synthesized through a facile sol–gel method followed by carbonization treatment, exhibiting superior sodium‐ion storage performance. The assembled NFP‐1.8//Bi@NC‐MF full‐cell also delivers an extraordinary cycling life.

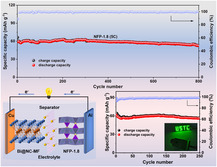

## 1 Introduction

Energy storage and conversion is a significant major challenge in the 21st century. Large‐scale energy storage devices are necessary to efficiently utilize renewable and clean energy, such as solar and wind energy.^[^ ^1^, ^2^ ^]^ Lithium‐ion batteries (LIBs) represent cutting‐edge technology for electric energy storage. However, the gradually consumed lithium reserves and corresponding soaring price stifle its further development, compelling scientists to burgeon more suitable alternatives.^[^ ^3^, ^4^, ^5^ ^]^ Under this background, sodium‐ion batteries (SIBs) have emerged as promising next‐generation alternatives, attracting increasing scientific interest.^[^ ^6^, ^7^, ^8^, ^9^, ^10^ ^]^ So far, developing suitable host materials that can adequately accommodate the rapid and reversible Na‐ion insertion–extraction is still the key to promoting the development of SIBs.^[^ ^11^, ^12^ ^]^

Among various low‐cost and sustainable cathodes, polyanionic transition‐metal compounds (phosphates,^[^ ^13^ ^]^ fluorophosphates,^[^ ^14^ ^]^ pyrophosphates,^[^ ^1^ ^]^ etc.) with excellent structural stability have carved out a niche for themselves since the discovery of LiFePO_4_ as the host matrix for Li‐ion insertion and extraction by Goodenough et al. in 1997.^[^ ^15^, ^16^, ^17^ ^]^ In particular, the cost‐effective and eco‐friendly NaFePO_4_, including the olivine and maricite structure, should be highlighted due to the high theoretical capacity (155 mA h g^−1^).^[^ ^18^, ^19^, ^20^ ^]^ Olivine NaFePO_4_, which is isomorphic to its Li‐counterpart of LiFePO_4_, is thermodynamically unstable and can only be produced by the electrochemical ion exchange route, hindering its practical application,^[^ ^21^, ^22^ ^]^ whereas the thermodynamically favored maricite NaFePO_4_ can be readily synthesized by the conventional synthetic routes.^[^ ^23^ ^]^ The difference between the two structures is mainly due to the occupying sites of Na^+^ and Fe^2+^. If the Fe^2+^ resides in the 4c site and the Na^+^ is located at the 4a site, the structure belongs to the olivine group, which has a 1D sodium‐ion diffusion channel. However, if the occupation of Na^+^ and Fe^2+^ is reversed, the structure belongs to the maricite group.^[^ ^24^, ^25^, ^26^ ^]^ Maricite NaFePO_4_ is generally considered electrochemically inactive due to its “closed” framework lacking Na^+^ diffusion pathways with low activation barriers.^[^ ^27^, ^28^, ^29^ ^]^ Nevertheless, at early 2015, Kang et al. first reported that all Na^+^ can be deintercalated from the nanosized maricite NaFePO_4_ with simultaneous transformation into amorphous FePO_4_, an unexpected capacity of 142 mA h g^−1^ (92% of the theoretical value) and an outstanding cyclability with 95% capacity retention after 200 cycles can be achieved.^[^ ^30^ ^]^ Various approaches, such as particle size reduction, coatings, and mesoporous structures, have been developed to improve electrochemical performance.^[^ ^31^ ^]^ Liu et al. reported the pioneering discovery that NaFePO_4_@C with minimum‐sized (1.6 nm) maricite NaFePO_4_ nanodots could deliver an intriguing reversible capacity of 145 mA h g^−1^ at 0.2 C and outstanding cyclic stability (≈89% capacity retention over 6,300 cycles).^[^ ^32^ ^]^ Su et al. also demonstrated that the highly dispersed maricite NaFePO_4_ nanoclusters with ultrafine NaFePO_4_@C subunits (3 nm) have excellent electrochemical performance, which delivers ultrahigh capacity (149.2 mA h g^−1^ at 0.2 C) and excellent rate performance (75.7 mA h g^−1^ at 50 C).^[^ ^33^ ^]^ All of the significant results mentioned above are related to the phase transition from maricite NaFePO_4_ to amorphous NaFePO_4_ during the charging process, whereas the electrochemical performance of maricite NaFePO_4_ is unsatisfactory.^[^ ^19^, ^21^, ^26^ ^]^ Consequently, developing an environmentally friendly synthesis method to improve the electrochemical performance of maricite NaFePO_4_ remains a practical challenge.

Based on the previous work, we report a cathode material of maricite NaFePO_4_ nanoparticles encapsulated within ultrathin carbon coating (m‐NFP@C) fabricated through a sol–gel and carbonization treatment. Due to their structural stability, ultrafine nanostructure, and uniform carbon coating, the electrodes deliver remarkable electrochemical performance in sodium ion half‐cells and full‐cells. Specifically, the obtained m‐NFP@C nanoparticles with 1.8 g polyvinyl pyrrolidone and annealed at 700 °C under N_2_ atmosphere (denoted as NFP‐1.8) exhibit a prominent capacity of 101.4 mA h g^−1^ with 90.5% capacity retention over 100 cycles at a current density of 20 mA g^−1^. Furthermore, the full‐cell with NFP‐1.8 as the cathode and the bismuth–carbon microfoam‐like compound (denoted as Bi@NC‐MF) as the anode exhibits remarkable cyclic stability over 250 cycles.

## 2 Results and Discussion

The m‐NFP@C nanoparticles are prepared through a facile sol–gel method followed by a calcination process. Briefly, the precursor powders first are prepared by a simple mixture of 1.8 g poly(vinylpyrrolidone) (PVP), equal molar ratios of NaH_2_PO_4_, Fe(NO_3_)_3_·9H_2_O, and citric acid in a certain amount of methanol and *N*,*N*‐dimethylformamide (DMF) solution followed by evaporation. The m‐NFP@C nanoparticles can then be generated by conventional thermal annealing treatment under N_2_ atmosphere at 700 °C (NFP‐1.8). To further explore the effects of PVP and annealing temperature, the m‐NFP@C nanoparticles with different PVP dosages (0, 0.9 and 2.7 g, denoted as NFP‐0, NFP‐0.9, and NFP‐2.7, respectively) and annealing temperature (500, 600, and 800 °C) are also fabricated as control samples. Detailed synthetic conditions are given in the experimental section of the Supporting Information.

**Figure 1:**
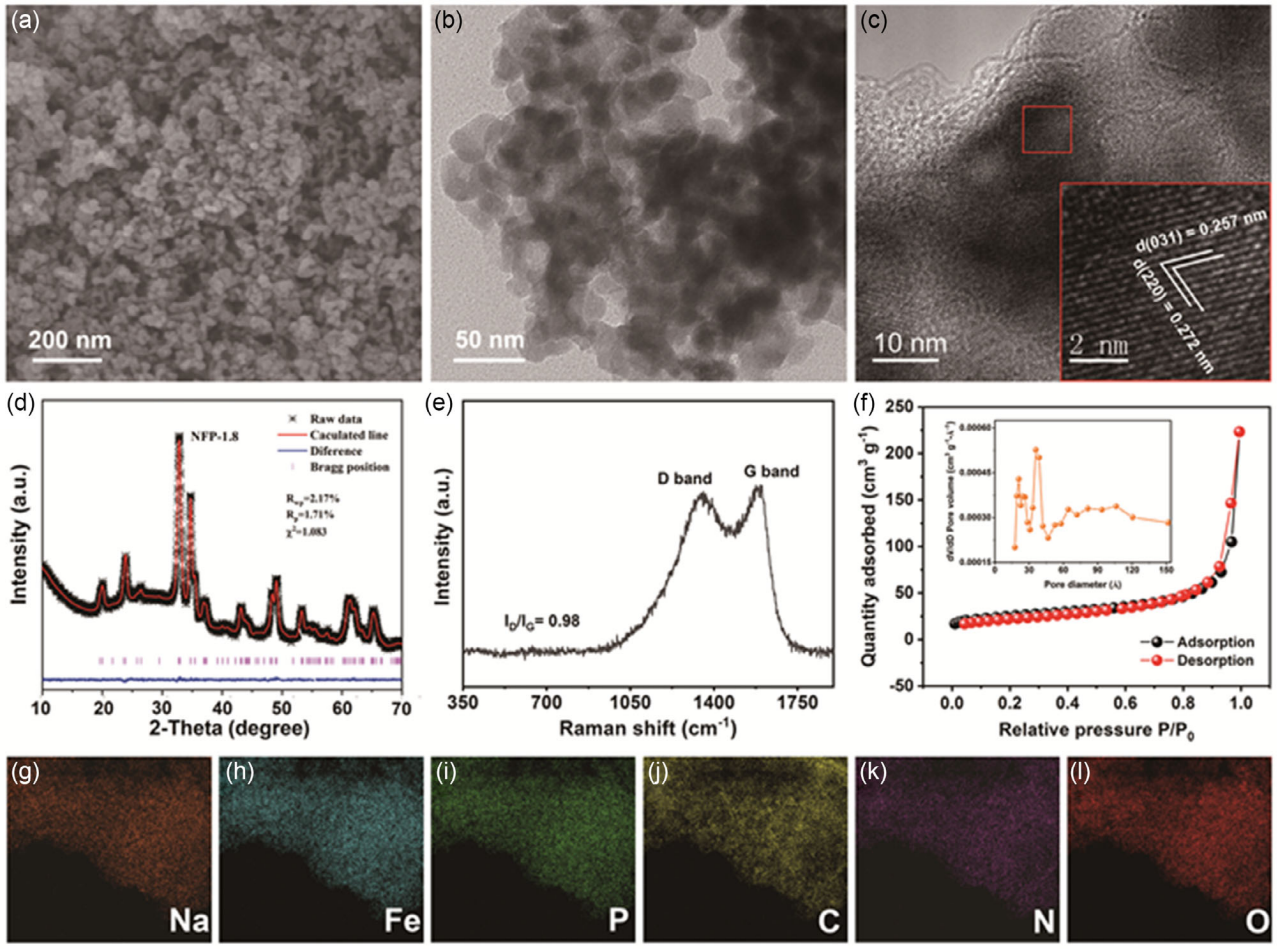
Morphological, microstructural, and compositional characterizations of NFP‐1.8. a) FESEM, b) TEM, and c) HRTEM images; d) Rietveld refinement XRD pattern; e) Raman spectrum; f) N_2_ adsorption‐desorption isotherm and pore size distribution curve (inset); and g–l) elemental mapping results.

The morphological and microstructural information of the as‐synthesized m‐NFP@C nanoparticles are observed by field‐emission scanning electron microscopy (FESEM) and transmission electron microscopy (TEM). The FESEM and TEM images (**Figure** **1**a,b) show that the NFP‐1.8 is composed of homogeneous and ultrafine nanoparticles, with average diameters of 30–50 nm. The microstructure of control samples is also investigated. Surprisingly, in the absence of PVP, the NFP‐0 exhibits a micronscale blocky structure (Figure S1, Supporting Information), which may be due to the aggregation of nanoparticles without the help of dispersants, whereas, excessive PVP (NFP‐2.7) also promotes the growth of nanoparticles (Figure S2b, Supporting Information). The above conditions are not conducive to the storage of sodium ions. Although the NFP‐0.9 is also composed of many nanoparticles (Figure S2a, Supporting Information), there is still aggregation of nanoparticles compared with the NFP‐1.8. What's more, whether the NFP‐1.8 is annealed at 500, 600, or 700 °C, it displays similar microstructure (Figure S3a–b, Supporting Information). Nevertheless, a floccule structure is produced when the annealing temperature is raised to 800 °C, as shown in Figure S3c, Supporting Information, which may be caused by overfiring of the material. Figure 1c displays the high‐resolution TEM (HRTEM) image of the NFP‐1.8; the nanoparticle is tightly wrapped by a carbon layer, which is advantageous for preventing volume change during cycling and improving electrical conductivity. The clear lattice fringes (inset of Figure 1c) manifest the good crystallinity and the interplanar spacings of 0.257 and 0.272 nm corresponding to the (031) and (220) planes of maricite NaFePO_4_, respectively. Furthermore, the associated X‐ray diffraction (XRD) pattern of NFP‐1.8 (Figure 1d) can be well indexed to maricite NaFePO_4_ (JCPDS No. 29‐1216) with the Pmnb space group. It should be noted that all diffraction peaks of all control samples can be commendably indexed to maricite NaFePO_4_, except the NFP‐1.8 annealed at 800 °C and the NFP‐0 (Figure S4, Supporting Information), for which the presence of impurity phases may be attributed to the pyrolysis of NaFePO_4_ and the incomplete reaction, respectively. Based on the above analysis, the dosage of PVP and the annealing temperature play crucial roles in preparing the uniform m‐NFP@C nanoparticles. Raman spectroscopy is measured to get more insights into the defect level in the carbon of the NFP‐1.8 sample. The dominated peaks centered at ≈ 1,350 and ≈ 1,590 cm^−1^ in Figure 1e are associated with the disordered and graphitic structures of carbon, respectively,^[^ ^34^ ^]^ further demonstrating the existence of carbon. As can be seen from Figure S5, Supporting Information, the peak intensity ratio of the D band to G band (*I*_D_/*I*_G_) of NFP‐1.8 varies with the annealing temperature, and the maximum value of 0.98 can be obtained when annealing at 700 °C, where more defects are involved in the carbon matrix. According to the thermogravimetric analyses (TGA) curves shown in Figure S6, Supporting Information, the weight ratio of carbon in NFP‐1.8 is determined to be about 20.1%. In contrast, a small amount of carbon in NFP‐0 may come from citric acid. To investigate the surface properties of the NFP‐1.8, we also carry out Brunauer–Emmett–Teller (BET) measurements, as shown in Figure 1f. The average pore size of NFP‐1.8 is about 3 nm, and the measured BET‐specific surface area is 84.1 m^2^ g^−1^, which are conducive to the accessibility of electrolyte and the rapid migration of Na^+^. More importantly, the high‐angle annular dark‐field scanning TEM (HAADF‐STEM) image (Figure S7, Supporting Information) and the corresponding elemental mapping images (Figure 1g–l) of the NFP‐1.8 directly confirm that Na, Fe, P, C, N, and O elements are uniformly distributed throughout the entire sample.

**Figure 2:**
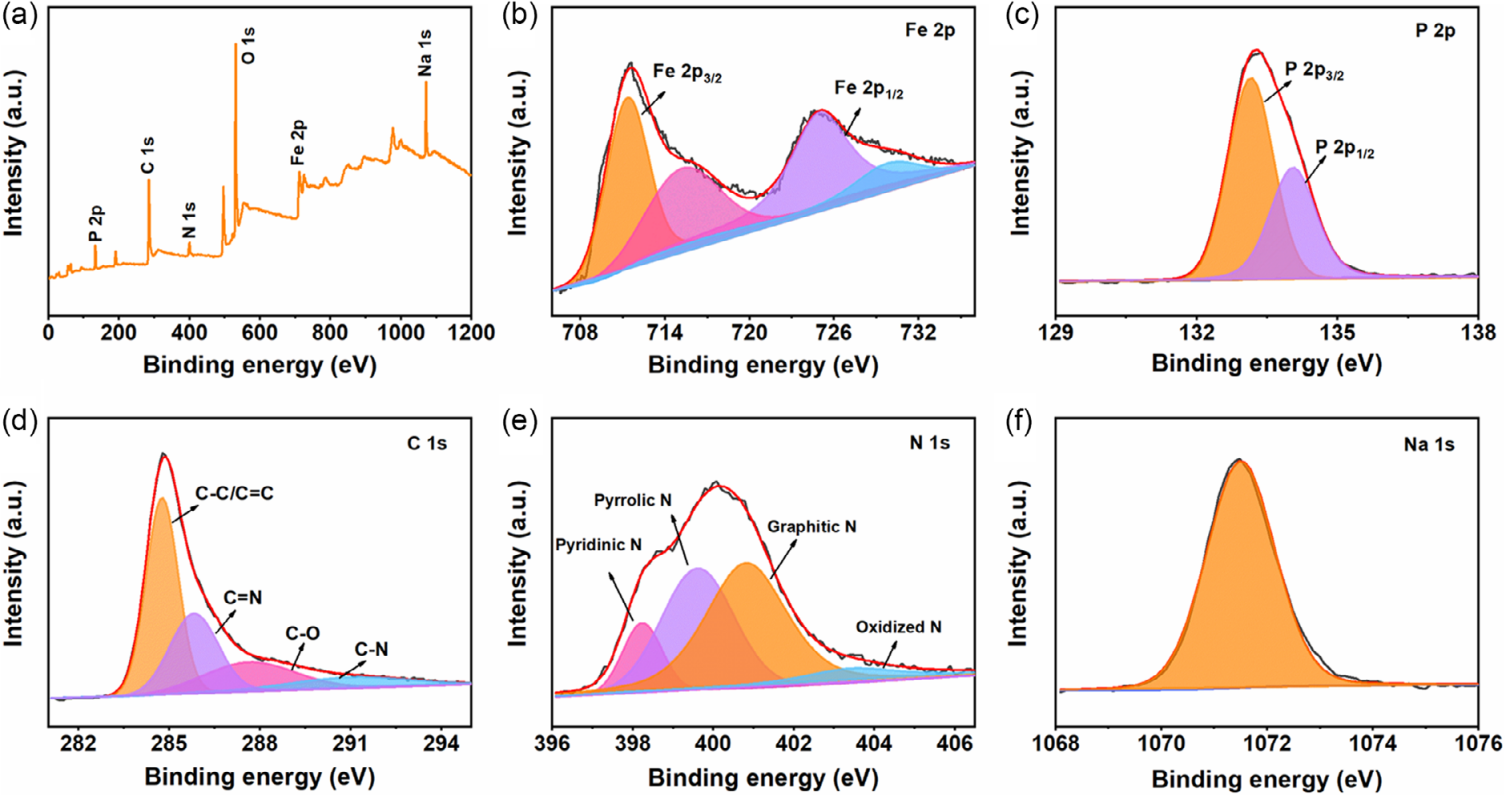
XPS spectra of the NFP‐1.8. a) Survey spectrum; and b–f) high‐resolution spectra of Fe 2p (b), P 2p (c), C 1s (d), N 1s (e), and Na 1s (f), respectively.

The composition and surface chemical states of the NFP‐1.8 are further measured by X‐ray photoelectron spectroscopy (XPS), as shown in **Figure** **2**. The XPS full spectrum in Figure 2a intuitively exhibits the presence of Na, Fe, P, C, N, and O elements, consistent with the above elemental mapping results. Figure 2b shows the high‐resolution Fe 2p XPS spectrum, which can be deconvoluted into four peaks. The two apparent peaks at 724.9 and 711.3 eV can be assigned to Fe 2p_1/2_ and Fe 2p_3/2_, respectively, indicating the existence of Fe^2+^.^[^ ^33^, ^35^ ^]^ In addition, the other two peaks at 729.8 and 715.3 eV are satellite peaks originating from the partially filled d‐orbitals of Fe.^[^ ^36^ ^]^ The high‐resolution P 2p XPS spectrum (Figure 2c) with two characteristic peaks at 134.05 eV (P 2p_1/2_) and 133.15 eV (P 2p_3/2_) manifests P^5+^ in PO_4_^3−^.^[^ ^37^ ^]^ Meanwhile, the high‐resolution C 1s XPS spectrum (Figure 2d) can be deconvoluted into four peaks, which can be ascribed to C–C /C = C (284.75 eV), C = N (285.8 eV), C–O (287.63 eV), and C–N (290.99 eV) bonds. C = N and C–N bonds showed the successful doping of heteroatoms in carbon.^[^ ^32^ ^]^ The high‐resolution XPS spectrum of N 1s (Figure 2e) contains four peaks referring to pyridinic N (398.23 eV), pyrrolic N (399.61 eV), graphitic N (400.81 eV), and oxidized N (403.41 eV) species, respectively. Notably, pyridinic N and pyrrolic N could absorb Na^+^ as active electrochemical sites through surface induced capacitance process. At the same time, graphitic N is conducive to improve the electron conductivity of the NFP‐1.8 through its electron donor characteristics.^[^ ^38^ ^]^ Furthermore, the Na 1s peak (Figure 2f) is observed at a binding energy of 1,071.5 eV and highly agrees with Na^+^.^[^ ^37^ ^]^ As demonstrated above, the m‐NFP@C nanostructure of homogeneous and ultrafine nanoparticles has been successfully prepared.

**Figure 3:**
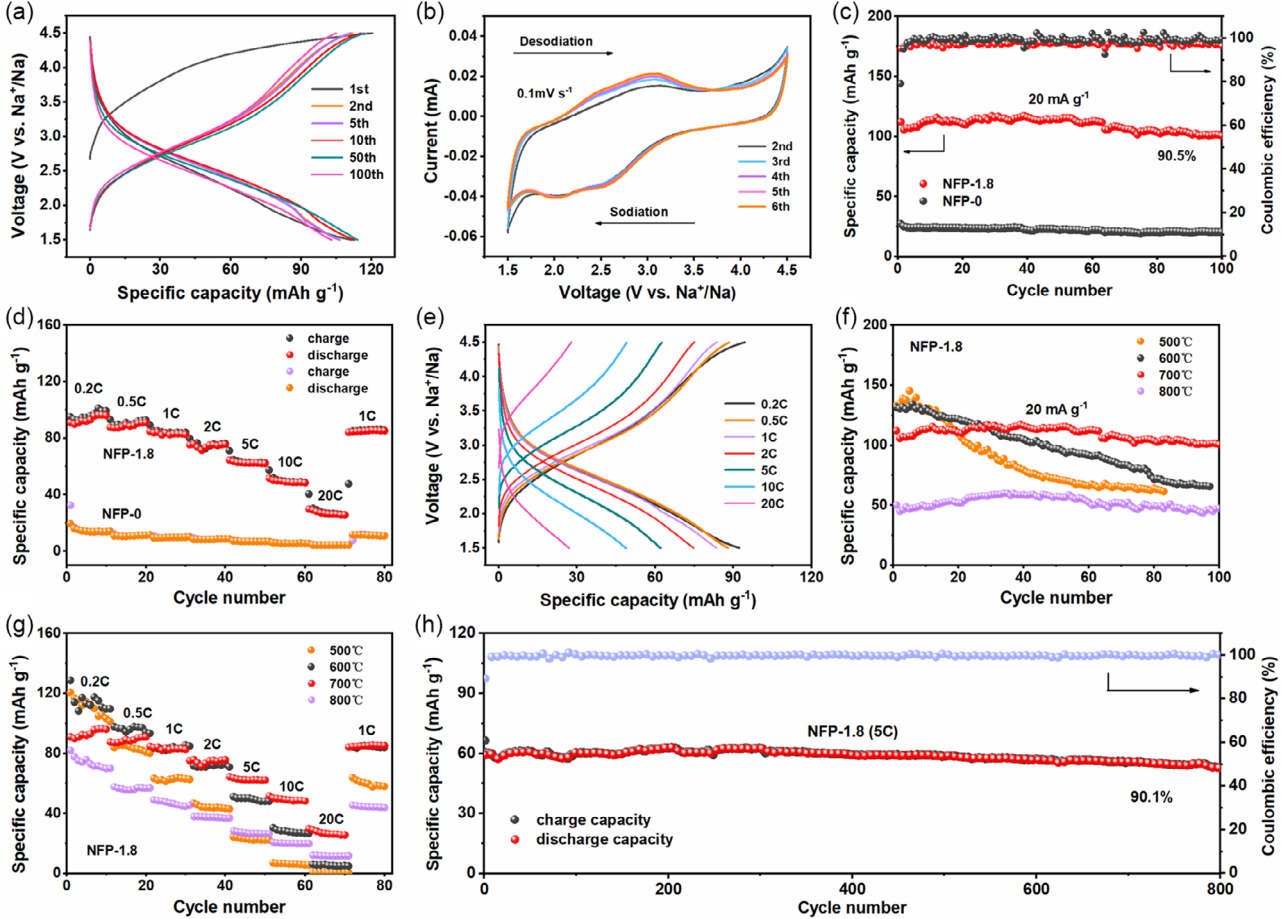
a–e) Sodium‐ion storage performance of the NFP‐1.8 cathode. a) GCD curves at a current density of 20 mA g^−1^; b) cyclic voltammetry curves at a scan rate of 0.1 mV s^−1^; c) cycling stability of the NFP‐1.8 and NFP‐0 at a current density of 20 mA g^−1^; d) rate performance of the NFP‐1.8 and NFP‐0 at various current densities from 0.2–20 C; e) charge–discharge curves of the NFP‐1.8 at various current densities from 0.2 to 20 C. f–h) Comparison of electrochemical performance of NFP‐1.8 composites at different annealing temperatures. f) Cycling stability at a current density of 20 mA g^−1^ and g) rate performance at various current densities. h) Long‐term cycling stability of the NFP‐1.8 at a current density of 5 C for 800 cycles.

The sodium storage performance of the NFP‐1.8 electrode is then evaluated with coin cells in half‐cell configuration with sodium foil as counter and reference electrodes in the voltage window of 1.5–4.5 V (vs Na^+^/Na). **Figure** **3**a depicts the galvanostatic charge‐discharge (GCD) profiles of the NFP‐1.8 electrode for the 1st, 2nd, 5th, 10th, 50th, and 100th cycles at 20 mA g^−1^. During the 1st cycle, the charge and discharge specific capacities of the NFP‐1.8 are 120.5 and 112.1 mA h g^−1^, respectively, corresponding to an initial coulombic efficiency as high as 93%. From the 2nd cycle onward, no obvious voltage plateau appears in the charge–discharge curves of different cycles, indicating no phase transition in the subsequent electrochemical reaction process.^[^ ^37^ ^]^ The representative cyclic voltammetry (CV) curves of NFP‐1.8 tested at a scan rate of 0.1 mV s^−1^ are used to investigate the reaction mechanism. As indicated by Figure 3b, a pair of current peaks located at 2.9 and 2.1 V can be observed, corresponding to the deintercalation–intercalation of Na^+^ with Fe^2+^ ↔ Fe^3+^.^[^ ^32^ ^]^ The broad redox peaks signify a continuous single‐phase sedation–dissociation reaction, keeping with the charge–discharge curves.^[^ ^39^ ^]^ Notably, there is considerable overlap between the CV curves, implying its good cycling stability. For demonstration purposes, the cycling stability of the NFP‐1.8 and NFP‐0 electrodes is comparatively evaluated at 20 mA g^−1^ (Figure 3c). In the first few cycles, the specific capacity fading of the composite electrode is mainly due to electrolyte decomposition and solid electrolyte interface (SEI) film formation. Then the specific capacity experiences a slight rise, which may be ascribed to the activation of the electrode material. For the NFP‐1.8 electrode, the reversible specific capacity is well retained at 101.4 mA h g^−1^ after 100 cycles with 90.5% capacity retention, which is prominent compared to previously reported results for NaFePO_4_ (Table S1, Supporting Information). While the NFP‐0 electrode can only maintain an inadequate capacity of 20.2 mA h g^−1^ after 100 cycles. Meanwhile, we also fully explore the effect of the variation of PVP amount on electrochemical performance, as shown in Figure S8, Supporting Information. Both NFP‐0.9 and NFP‐2.7 electrodes show excellent cycling stability, but the specific capacity of NFP‐2.7 is relatively low, illustrating that too much PVP is detrimental to the electrochemical performance. Besides, the electrochemical performance of PVP was also explored with nearly negligible electrochemical capacity, as shown in Figure S9, Supporting Information. In addition, the cycling performance comparison of NFP‐1.8 and NFP‐0.9 at a current density of 1 C (1 C = 155 mA g^−1^) is further provided in Figure S10 and S11, Supporting Information, the NFP‐1.8 retains outstanding capacities than NFP‐0.9 whether before or after 250 cycles (99.5 vs 77.3 and 88.1 vs 86.5 mA h g^−1^, respectively). Furthermore, the rate capabilities of the NFP‐1.8 and NFP‐0 electrodes are also investigated with current densities ranging from 0.2 to 20 C (Figure 3d). For NFP‐1.8, the average discharge capacities are 93.4, 89.1, 83.5, 75.3, 62.8, 49.7, and 27.1 mA h g^−1^ at the stepwise increased current densities of 0.2, 0.5, 1, 2, 5, 10, and 20 C, respectively. Despite the high‐rate charge–discharge cycling, the NFP‐1.8 can still output an outstanding reversible capacity of 85.3 mA h g^−1^ when the current density is reduced to 1 C. In contrast, the NFP‐0 delivers unsatisfactory discharge capacities at all current densities. Figure 3e displays the charge–discharge profiles of the NFP‐1.8 at various current densities. The discharge capacities decrease gradually with increasing current rates, which may be attributed to the increased polarization.^[^ ^22^ ^]^

To further investigate the influence of annealing temperature on electrochemical performance, we systematically compare the cycling stability and rate performance of the NFP‐1.8 composite under different annealing temperatures (500, 600, 700, and 800 °C). As depicted in Figure 3f,g, the surprisingly high discharge capacities of 132.9 and 131.4 mA h g^−1^ (85.7% and 84.8% of the theoretical capacity, respectively) are achieved at 20 mA g^−1^ under the annealing temperature of 500 and 600 °C. However, the fast capacity decay and poor rate performance hinder its further research. When raising the annealing temperature to 800 °C, only a specific capacity of 47.4 mA h g^−1^ is retained after 100 cycles at a current density of 20 mA g^−1^ and the rate performance is also inferior, which may be due to the presence of impurity phase. It can be seen from the above results that the selection of the appropriate PVP amount and annealing temperature plays a crucial role in the electrochemical performance of maricite NaFePO_4_. Here, 1.8 g PVP and 700 °C annealing are the best choices. Finally, as an important descriptor for electrode materials, the long‐term cycling stability of the NFP‐1.8 for Na^+^ storage is then researched under a current density of 5 C (Figure 3h). As can be seen, a superior specific capacity of 53.3 mA h g^−1^ can be maintained after 800 cycles with coulombic efficiency of around 100%. More astonishingly, a remarkable capacity retention of 90.1% can still be achieved after long‐term cycling. In short, the superior rate capacity and longer cycling stability of the NFP‐1.8 make it possible to be used in large‐scale energy storage devices.

**Figure 4:**
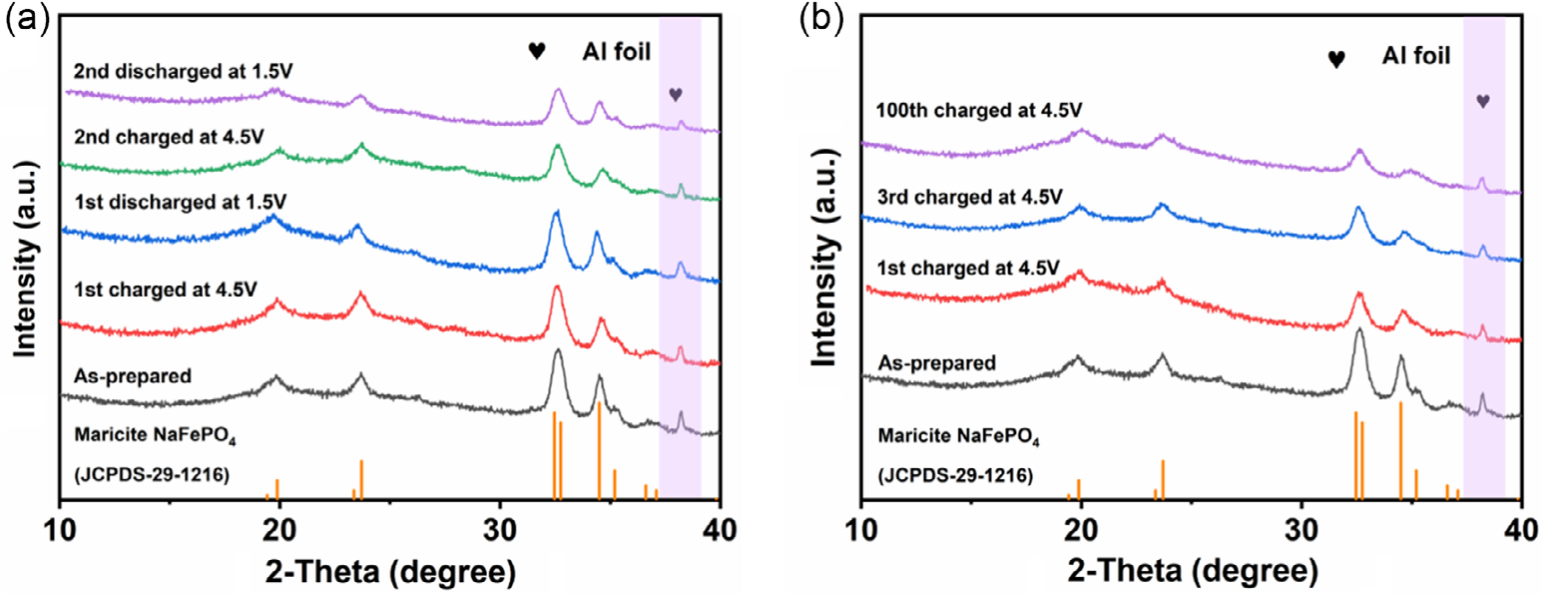
a) Ex situ XRD patterns of the NFP‐1.8 electrode at the fully charged (4.5 V) and discharged (1.5 V) states in the first two cycles. b) Ex situ XRD patterns of the NFP‐1.8 electrode at the fully charged (4.5 V) states in the 1st, 3rd, and 100th cycles.

Ex situ XRD is carried out to better understand the structural change of the NFP‐1.8 cathode during cycling. **Figure** **4**a exhibits the ex situ XRD patterns of the NFP‐1.8 electrode at the fully charged (4.5 V) and discharged (1.5 V) states in the first two cycles, and the XRD pattern of the pristine NFP‐1.8 is also given for comparison purpose. Astonishingly, all the peaks belonging to the maricite structure remain unchanged during the sodium extraction–insertion process, indicating that amorphization is nonexistent during the cycles, which is in contrast to the previously reported phase transition from maricite NaFePO_4_ to amorphous NaFePO_4_ in the charging process.^[^ ^33^, ^37^ ^]^ Besides, the structural volume changes at different charge/discharge states in the first two cycles were investigated by the Rietveld refinement XRD patterns in Figure S12, Supporting Information and Table S2, Supporting Information, which could manifest small volume change. Meanwhile, we also investigate the in situ XRD of NFP‐1.8 for the first cycle, as shown in Figure S13, Supporting Information, which is consistent with the above results. To further verify the experimental results, the XRD patterns of the NFP‐1.8 electrode at the fully charged (4.5 V) states in the 1st, 3rd, and 100th cycles are compared with the as‐prepared NFP‐1.8, no significant peak shift is observed except the decrease of peak intensity. From these findings, it can be claimed that the NFP‐1.8 possesses excellent structural stability, contributing to its cycling performance.

**Figure 5:**
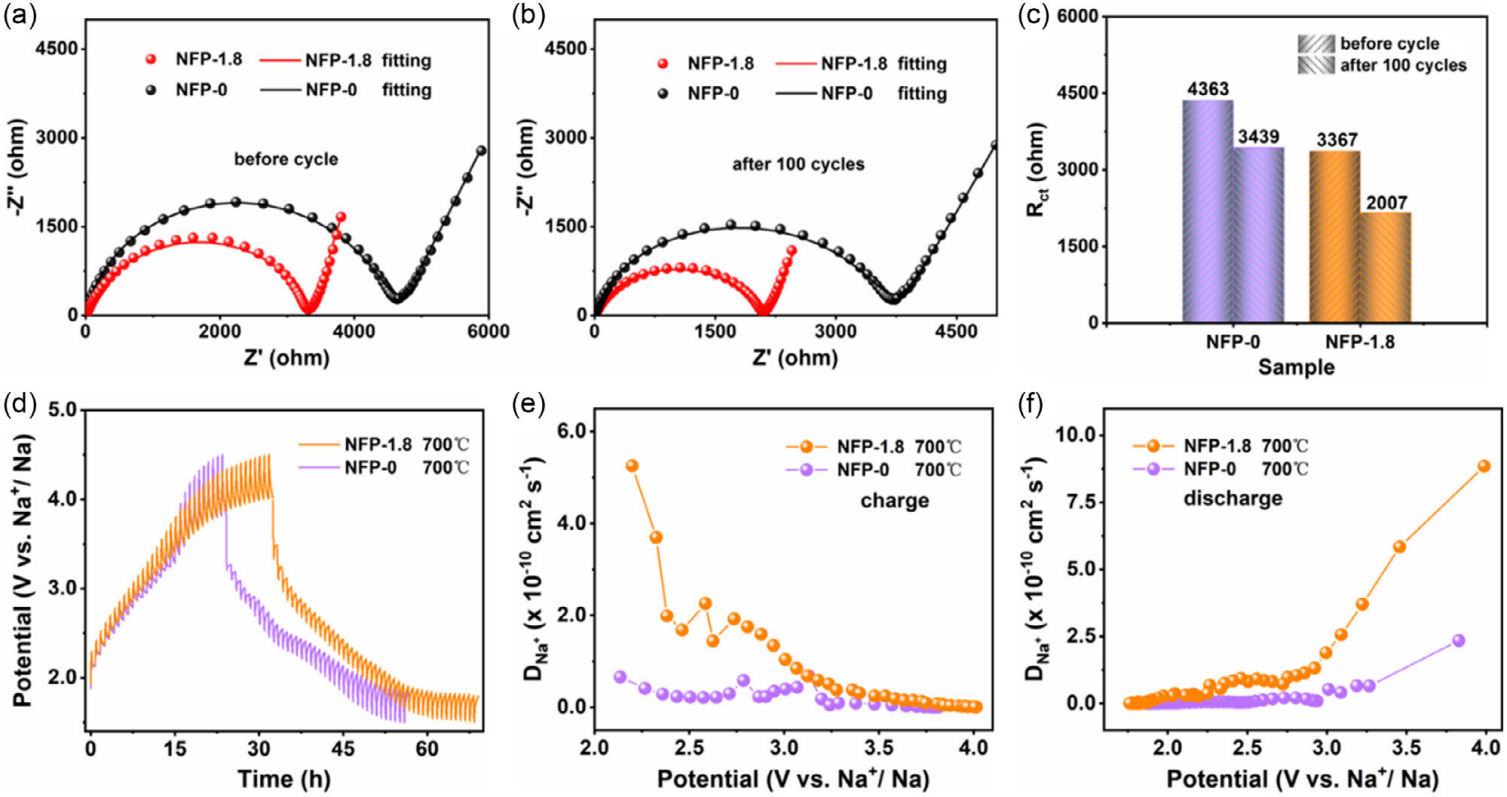
Kinetics analysis. a,b) EIS results of the NFP‐1.8 and NFP‐0 electrodes before cycling (a) and after 100 cycles (b). c) Comparison of *R*_ct_ values between the NFP‐1.8 and NFP‐0 electrodes. d) GITT curves of the NFP‐1.8 and NFP‐0 electrodes. e,f) Calculated Na^+^ diffusion coefficients of the charge (e) and discharge (f) processes.

Kinetic analysis is investigated to unravel the possible origins of the enhanced electrochemical performance in‐depth, as shown in **Figure** **5**. First, CV curves of the NFP‐1.8 and NFP‐0 electrodes recorded at various scan rates ranging from 0.1 to 1.0 mV s^−1^ are exhibited in Figure S14a and S15, Supporting Information. The excellent linear fitting results (Figure S14b, Supporting Information) of the peak currents and the square root of scan rates (*v* ^1/2^) suggest that the electrochemical Na^+^ extraction–insertion reaction is a diffusion‐controlled process. Then, the electrochemical impedance spectra (EIS) measurements of the NFP‐1.8 and NFP‐0 electrodes are also employed before the first cycle and after 100 cycles (Figure 5a,b). The semicircle in the high‐ and middle‐frequency regions and the straight line in the low‐frequency region represent the charge transfer process on the electrode surface and the diffusion process of sodium ions in the electrode, respectively.^[^ ^20^ ^]^ The equivalent circuit is also shown in Figure S16, Supporting Information, where *R*_s_ is the solution resistance, CPE and *R*_ct_ represent the constant‐phase element, and charge transfer resistance, respectively, while *Z*_w_ corresponds to Warburg impedance.^[^ ^24^, ^40^ ^]^ It is worth noting that the *R*_ct_ is a key indicator of the electrodes for the charge transfer kinetics. According to the fitting results (Figure 5c), the NFP‐1.8 electrode exhibits a much smaller *R*_ct_ than the NFP‐0 electrode whether before or after cycles (3367 vs 4363 and 2007 vs 3439 Ω, respectively), indicating that the nanosized active materials provide an easier transport of Na^+^ through the electrode–electrolyte interface, which can effectively improve the electrochemical performance. Additionally, the galvanostatic intermittent titration technique (GITT) is further performed to intuitively investigate the Na^+^ diffusion kinetic, whose curves are displayed in Figure 5d and Figure S17a,b, Supporting Information. It is generally adopted that the Na^+^ diffusion coefficient can be calculated from the GITT potential profiles using Fick's second law with the following equation

(1) $$D_{{\text{Na}}^{+}}=\frac{4}{\pi \tau }{\left(\frac{m_{B}V_{M}}{M_{B}S}\right)}^{2}{\left(\frac{\Delta E_{s}}{\Delta E_{\tau }}\right)}^{2},\tau <<\frac{L^{2}}{D}$$

where *D*_Na_^+^ (cm^2^ s^−1^) means the Na^+^ diffusion coefficient, *τ* denotes the constant current pulse time, and *m*_B_, *M*_B,_ and *V*_M_ represent the mass loading, the molar mass, and the molar volume of the active material, respectively. S is the surface area of the electrode (1.13 cm^2^), while Δ*E*_S_ and Δ*Eτ* (V) correspond to the quasi‐equilibrium potential and the potential difference during the current pulse, respectively.^[^ ^41^, ^42^, ^43^ ^]^ Visibly, the calculated *D*_Na_^+^ of the NFP‐1.8 electrode is far higher than that of control samples (Figure 5e,f and Figure S17c,d, Supporting Information) whether during the charge or discharge process, implying more favorable Na^+^ extraction–insertion kinetics owing to the unique structure of m‐NFP@C nanoparticles, which is consistent with the results of EIS. In a word, it can be concluded that downsizing to the nanoscale and the introduction of carbon coating could indeed promote the reaction kinetics for the maricite NaFePO_4_ compounds, which is responsible for the enhanced cycling capability and rate performance.

**Figure 6:**
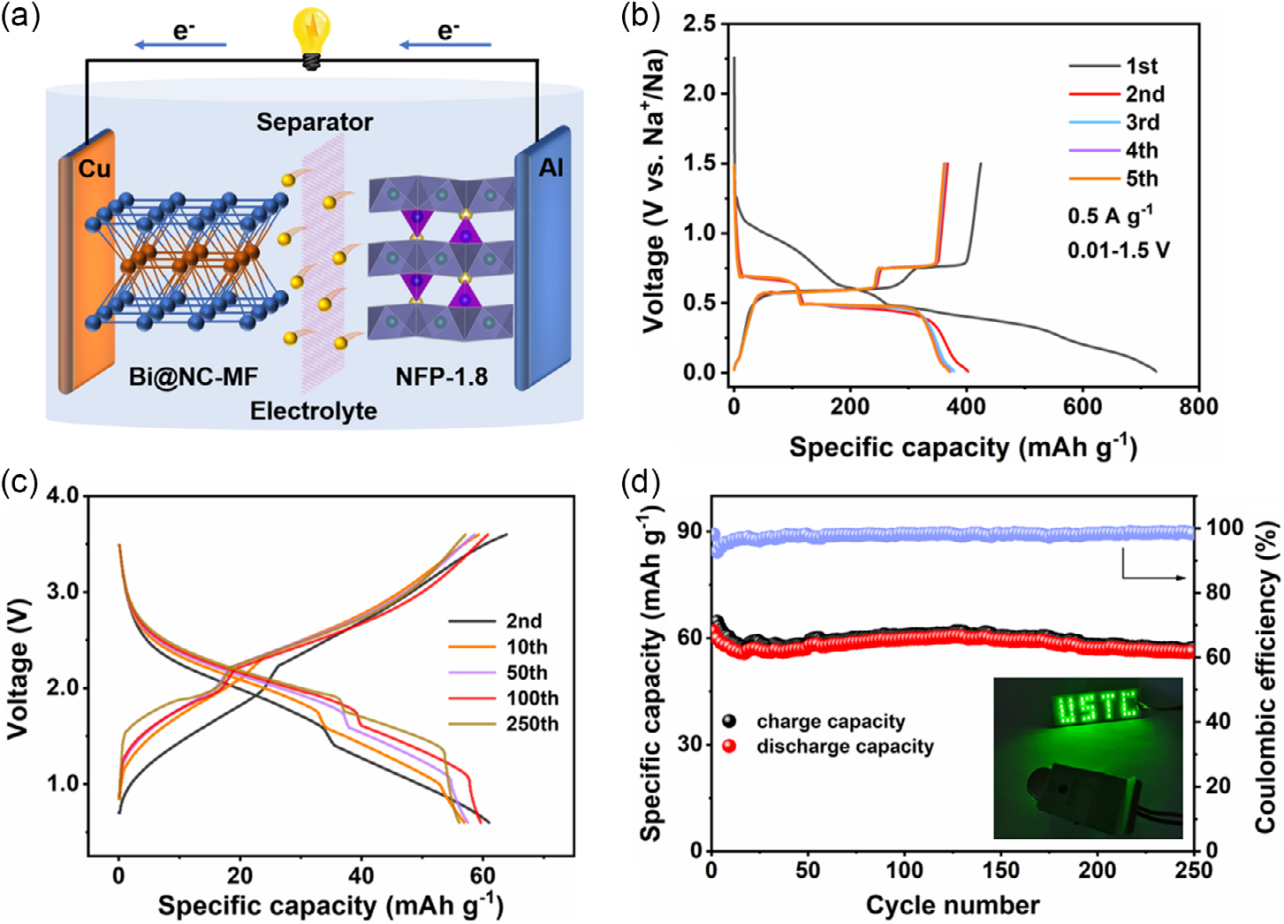
Evaluations on the full‐cell device based on NFP‐1.8 cathode and Bi@NC‐MF anode. a) Schematic illustration; b) charge–discharge curves of Bi@NC‐MF; c) charge–discharge curves, and d) cycling performance of the NFP‐1.8//Bi@NC‐MF full‐cell at 0.5 C in the voltage window of 0.6–3.6 V (based on the cathodic mass, the inset digital photo is a “USTC” logo consisting of 32 green LEDs powered by the full‐cell).

To verify the practical application feasibility of the as‐prepared NFP‐1.8 composite, the sodium‐ion full‐cell (SIFC) based on NFP‐1.8 cathode and Bi@NC‐MF anode (denoted as NFP‐1.8//Bi@NC‐MF) is further fabricated according to the configuration schematically illustrated in **Figure** **6**a. The GCD curves of the Bi@NC‐MF anode for the first five cycles at 0.5 A g^−1^ are shown in Figure 6b. The first discharge and charge capacities are 725.82 and 424.39 mA h g^−1^, respectively, delivering a low coulombic efficiency of 58.5%, which will undoubtedly negatively impact the full‐cell performance. Therefore, before the assembly of the SIFC, the Bi@NC‐MF anode is preactivated in half‐cell for 5 cycles to form a stable SEI film and reduce the irreversible capacity loss.^[^ ^34^ ^]^ In addition, considering the specific capacity difference between the cathode and the anode materials, the mass ratio between the two electrodes is carefully regulated and controlled based on the charge balance principle of the full‐cell device.^[^ ^44^ ^]^ Figure 6c displays the typical charge–discharge curves of the SIFC for the 2nd, 10th, 50th, 100th, and 250th cycles at 0.5 C within the voltage window of 0.6–3.6 V. Notably, the SIFC shows an average output voltage of around 1.99 V. More impressively, the reversible capacity remains at 56.2 mA h g^−1^ after 250 cycles with a high coulombic efficiency (CE) of over 98%, affording a remarkable capacity retention of 90.6% (Figure 6d). To further demonstrate the practicability of NFP‐1.8 composites, a “USTC” logo consisting of 32 green light‐emitting diodes (LEDs) can be continuously lighted up by the SIFC (inset, Figure 6d). In brief, all these results reveal that our strategy of homogeneous and ultrafine m‐NFP@C nanoparticles has a great prospect for practicability as a reliable sodium cathode candidate.

## 3 Conclusion

We have successfully developed an effective strategy to achieve dramatic improvement in the electrochemical performances of maricite NaFePO_4_ after a systematic study on the effects of PVP and annealing temperature. The synthesized NFP‐1.8 material demonstrates an impressive reversible capacity of 101.4 mA h g^−1^ at a current density of 20 mA g^−1^ with capacity retention up to 90.5% after 100 cycles. The capacity increase is thoroughly examined through microstructure characterization and kinetics analysis. These investigations reveal that downsizing to the nanoscale can significantly reduce the diffusion pathways for Na^+^/electron. Additionally, incorporating carbon coating enhances the electronic conductivity of electrode materials and effectively prevents nanoparticle aggregation and fragmentation. More impressively, the sodium‐ion full cell assembled with NFP‐1.8 cathode and Bi@NC‐MF anode also exhibits remarkable cycling performance with 90.6% capacity retention over 250 cycles. This remarkable achievement not only signifies a significant advancement in the practical application of high‐performance maricite NaFePO_4_, but also highlights the transformative potential of employing chemical methods to activate traditionally inactive materials.

## Conﬂict of Interest

The authors declare no conﬂict of interest.

## Supporting information

Supplementary Material

## Data Availability

The data that support the findings of this study are available from the corresponding author upon reasonable request.
