# Supplementary material for: Co‐Manipulation of Ultrafine Nanostructure and Uniform Carbon Layer Activates Maricite‐Structured NaFePO4 as a High‐Performance Cathode for Sodium‐Ion Batteries
Source: Small Sci. 2023 Nov 20;3(12):2300122. doi: 10.1002/smsc.202300122 (PMC11935972; doi:10.1002/smsc.202300122)
Supplement: Supplementary file 1 — Supplementary Material [file SMSC-3-2300122-s001.pdf]

Supporting Information

Co-Manipulation of Ultrafine Nanostructure and Uniform Carbon Layer Activates Maricite-Structured $NaFePO_4$ as a High-Performance Cathode for Sodium-Ion Batteries

Liping Zhao[+,a] *Lai Yu*[+,a] *Guanglin Wan*[+,a] *Nazir Ahmad,*[a] *Xinyi Ma,*[a] *Zongzhi Tao*[a] *and Genqiang Zhang*[*,a]

Ms. L. P. Zhao, Mr. L. Yu, Mr. G. L. Wan, Mr. N. Ahmad, Ms. X. Y. Ma, Mr. Z. Z. Tao and Prof. G. Q. Zhang

[a]Hefei National Research Center for Physical Sciences at the Microscale, CAS Key Laboratory of Materials for Energy Conversion, Department of Materials Science and Engineering, University of Science and Technology of China, Hefei, Anhui 230026, China

* To whom the correspondence should be referred. Email: gqzhangmse@ustc.edu.cn

+ These authors contribute equally to this work.

1. Experimental section:

1.1 Materials

All the chemicals were used directly after purchase without further treatment. Iron(III) nitrate nonahydrate ($Fe(NO_3)_3{\cdot}9H_2O$), Bismuth nitrate pentahydrate ($Bi(NO_3)_3{\cdot}5H_2O$), Sodium dihydrogen phosphate anhydrous (NaH_2PO_4), citric acid, N,N-Dimethylformamide (DMF), Hexamethylenetetramine (HMT), methanol, ethanol, carbon black (Super-P) and polyvinylidene fluoride (PVDF) were provided by Aladdin Industrial Corporation. Polyvinyl pyrrolidone (PVP) was purchased from Sinopharm Chemical Reagent Corporation.

1.2 Synthesis of maricite $NaFePO_4$@C nanoparticles:

The $NaFePO_4$@C nanoparticles were prepared by a sol-gel method followed by a calcination process. First, 20 ml of methanol and 10 ml of DMF were mixed to form a transparent solution. Next, 1.8 g PVP was added into the uniform solution with stirring for 1 h. Subsequently, $Fe(NO_3)_3{\cdot}9H_2O$ (6 mmol), as the iron source, NaH_2PO_4 (6 mmol), as the phosphorus and sodium sources, and citric acid (6 mmol), as the reducing agent, were successively added into the solution under vigorous magnetic stirring at room temperature until a transparent yellow solution was obtained. After that, the precursor solution was transferred to a drying oven and maintained at 80 °C overnight. The obtained dry precursor was collected and first heated at 300 °C for 2 h, then annealed at 700 °C for 3 h in the N_2 atmosphere with a heating rate of 2 °C min^{-1}. After naturally cooling to room temperature, the maricite $NaFePO_4$@C nanoparticles (denoted as NFP-1.8) were obtained. To explore the effects of PVP, we also prepared samples using various PVP amounts (such as 0, 0.9 and 2.7 g PVP, denoted as NFP-0, NFP-0.9 and NFP-2.7, respectively), while the other synthetic conditions remained unchanged. For further comparison, the NFP-1.8 samples with different annealing temperatures

were also investigated following the same preparation procedure by setting the annealing temperature to 500 °C, 600 °C and 800 °C, respectively.

1.3 Synthesis of Bi@NC-MF:

The hierarchical bismuth-carbon microfoam-like hybrid structure (denoted as Bi@NC-MF) was prepared according to our previous report. In a typical synthesis, 5 mmol of $Bi(NO_3)_3 \cdot 5H_2O$ and 10 mmol of HMT were dispersed in 50 mL of the ethanol solution, respectively. Then, the two obtained solutions were mixed rapidly with further continuous stirring for 3 h at room temperature, and milky precipitates of Bi-HMT coordination compounds were generated visibly. Subsequently, the precipitates were separated by vacuum filtration and dried in a vacuum oven at 60 °C overnight. Finally, the dry powder was collected and annealed in a tube furnace at 600 °C for 2 h with a heating rate of 2 °C min^{-1} in the Ar atmosphere. The Bi@NC-MF products were obtained after naturally cooling to room temperature.

2. Materials characterizations:

The morphologies and microstructures of the as-obtained samples were investigated by field-emission scanning electron microscopy (FESEM, SU-8200, Sirion200), transmission electron microscopy (TEM, Hitachi HT7700, JEM-2010), powder X-ray diffractor (XRD, Rigaku, MiniFlex 600, Ultima IV) and Raman spectrometer (Renishaw, 532 nm excitation laser). X-ray photoelectron spectroscopy (XPS, ESCALAB 250) was used to analyze the surface element and chemical valence. The particular surface area and pore size distribution of the obtained samples were evaluated through a chemisorption analyzer (Micrometritics, ASAP 2020). The composition of the samples was measured by thermogravimetric analysis (TGA, Mettler-Toledo TGA/DSC 1/1600) with a ramp rate of 10 °C min^{-1} under air atmosphere. *In-situ*

X-ray powder diffraction measurement was collected by using a specific modularized cell (Beijing Scistar Technology Co., Ltd) with a beryllium window for X-ray penetration.

3. Electrochemical measurements:

The electrochemical performance of the as-prepared materials was evaluated by assembling the CR2016 coin-type cells in an argon-filled glove box, where both the O_2 and H_2O concentrations were below 0.1 ppm. Specifically, the positive electrodes were fabricated by spreading the highly dispersed slurry (consisting of active materials, carbon black (Super-P) and polyvinylidene fluoride (PVDF) with a weight ratio of 7:2:1 in N-Methyl-2-Pyrrolidone (NMP) solvent) on the aluminum foils and drying at 100 °C for 12 h in an electric vacuum oven. The typical mass loading of the active materials was controlled to be about 2.0 mg cm^{-2}. As for the preparation of the anode electrode, Bi@NC-MF, Super-P and PVDF (the weight ratio 7:2:1) were also dispersed in NMP to form a uniform slurry, while the slurry was coated on copper foils, following dried at 100 °C for 12 h in an electric vacuum oven. Both the cathode and anode electrodes were cut into specific size (12 mm in diameter). When assembling the half-cells, the sodium foil was used as the counter electrode and reference electrode, the electrolyte was 1.0 M $NaClO_4$ in propylene carbonate with 5 vol% of fluoroethylene carbonate and the separator was glass microfiber filter (GF/F, Whatman). As to the full-cell device, it was assembled with NFP-1.8 as the cathode and Bi@NC-MF as the anode (denoted as NFP-1.8//Bi@NC-MF), the mass ratio of cathode/anode was controlled based on the charge balance principle. It should be mentioned that the Bi@NC-MF anode was pre-cycled for five cycles at a current density of 0.5 A g^{-1} in half-cells to eliminate the large irreversible capacity during the first charge-discharge processes. The separator was the same as those used in half-cells, while the electrolyte was 1 M $NaPF_6$ in ethylene glycol dimethyl ether (DME).

Cyclic voltammetry (CV) at different sweep rates from 1.5-4.5 V (*vs.* Na^+/Na) and electrochemical impendence spectra (EIS) from 100 kHz to 0.01 Hz were tested on the half-cells using a CHI660E electrochemical workstation. The galvanostatic charge-discharge tests, rate performance and galvanostatic intermittent titration technique (GITT) were performed on a NEWARE battery test system (CT-4008). Moreover, the NFP-1.8//Bi@NC-MF full-cells were galvanostatic charged-discharged in the voltage range of 0.6 and 3.6 V. The specific capacities of the half-cells and full-cells were calculated based on the weight of the NFP in the electrode. All the electrochemical tests were carried out at room temperature.

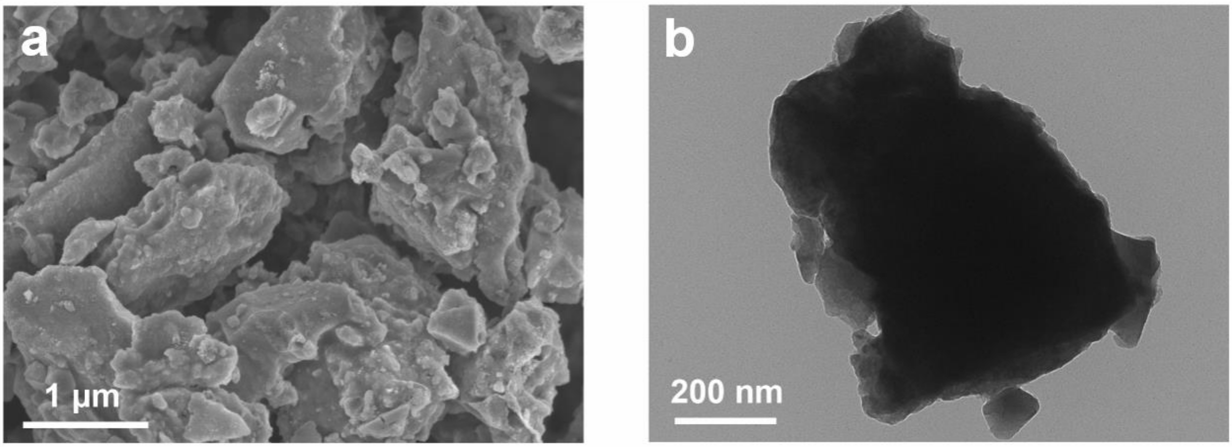

Figure S1. (a) FESEM and (b) TEM images of NFP-0.

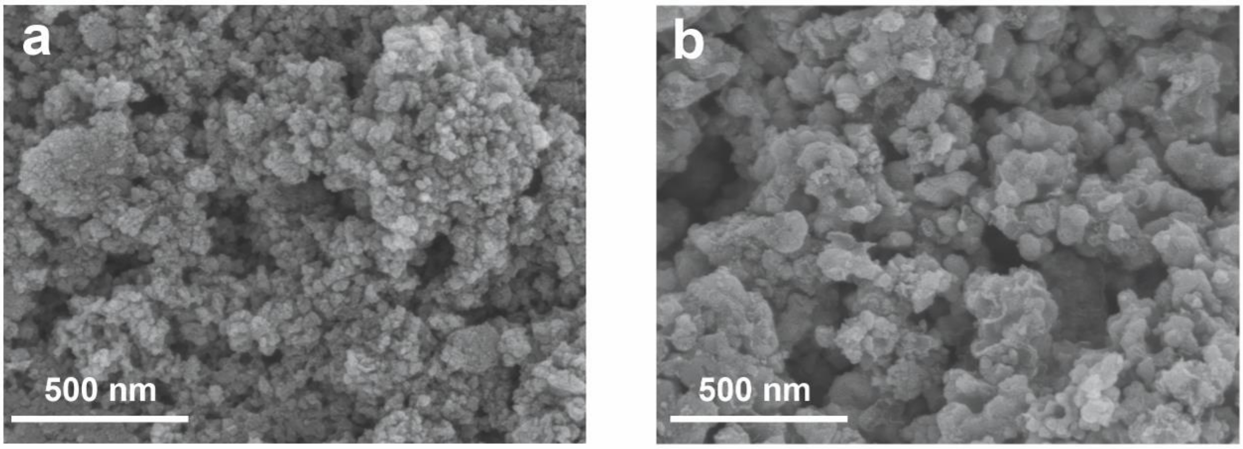

Figure S2. FESEM images of (a) NFP-0.9 and (b) NFP-2.7.

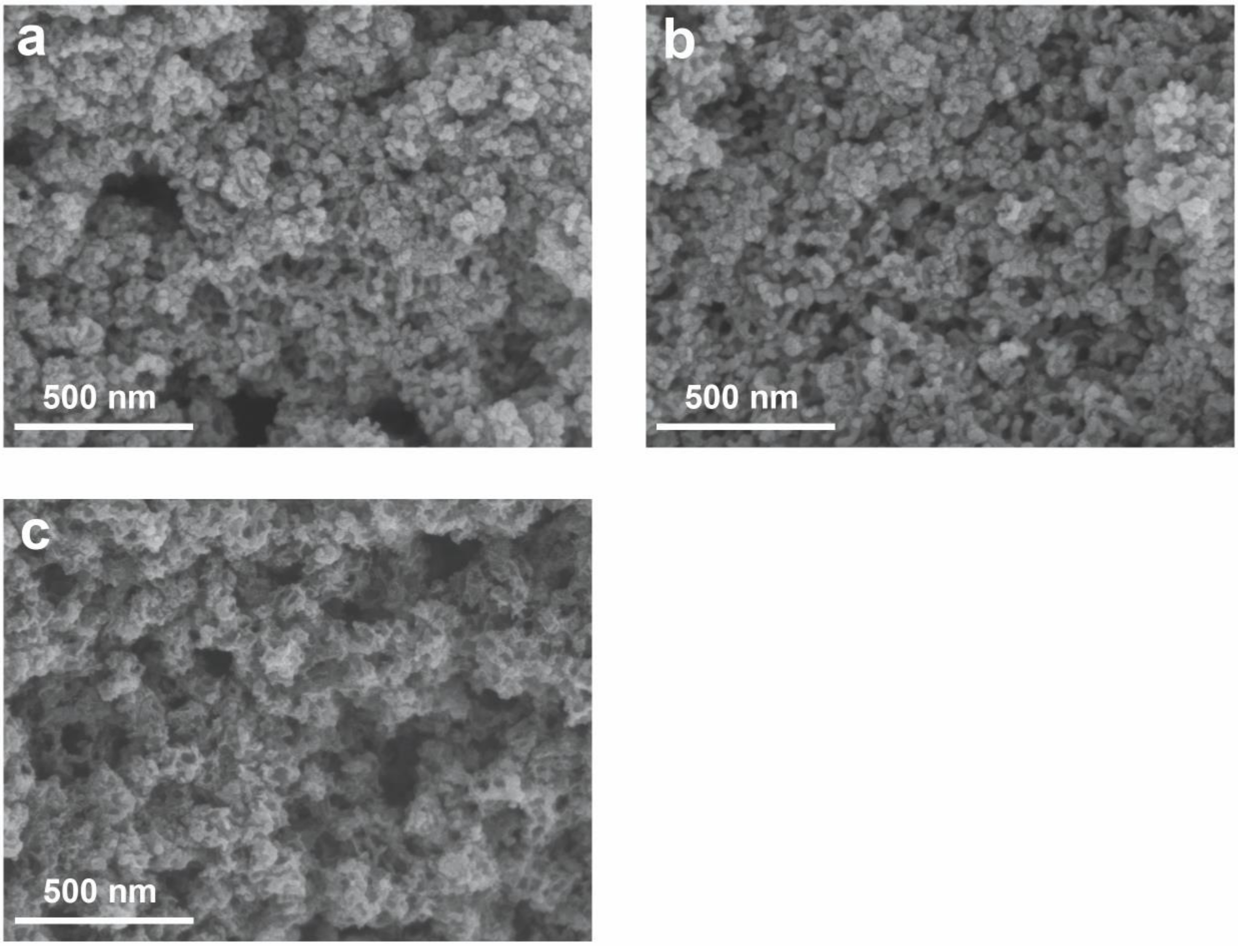

Figure S3. FESEM images of NFP-1.8 annealed at (a) 500°C, (b) 600°C and (c) 800°C.

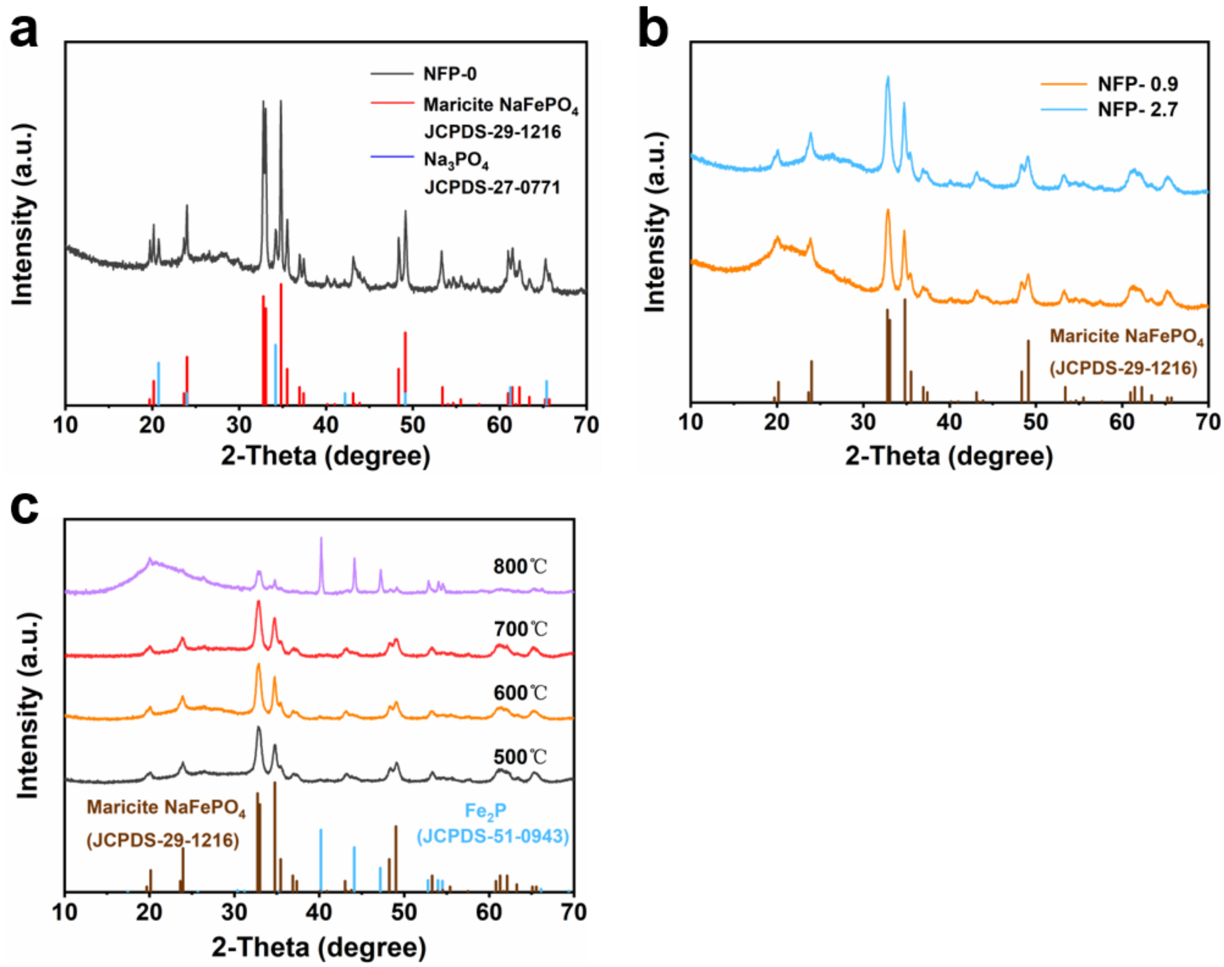

Figure S4. XRD patterns of (a) NFP-0; (b) NFP-0.9 and NFP-2.7; (c) NFP-1.8 at different annealing temperatures.

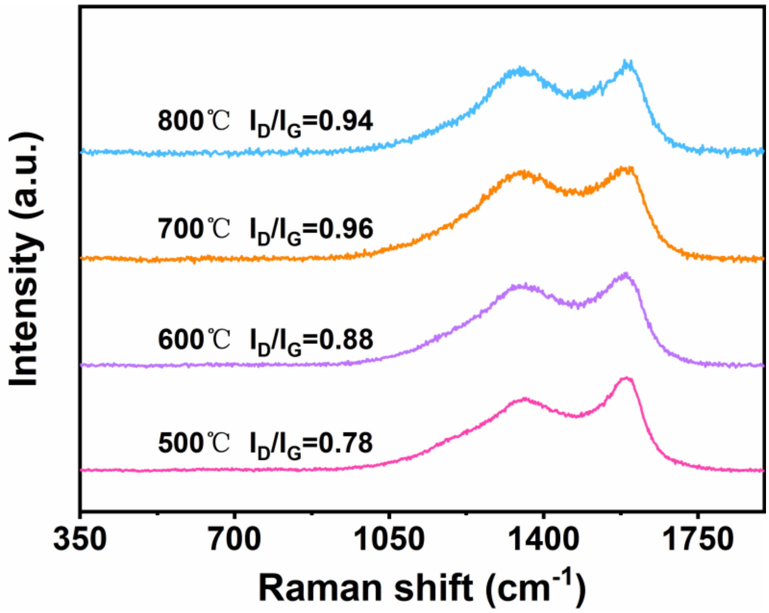

Figure S5. Raman patterns of NFP-1.8 annealed at different temperatures.

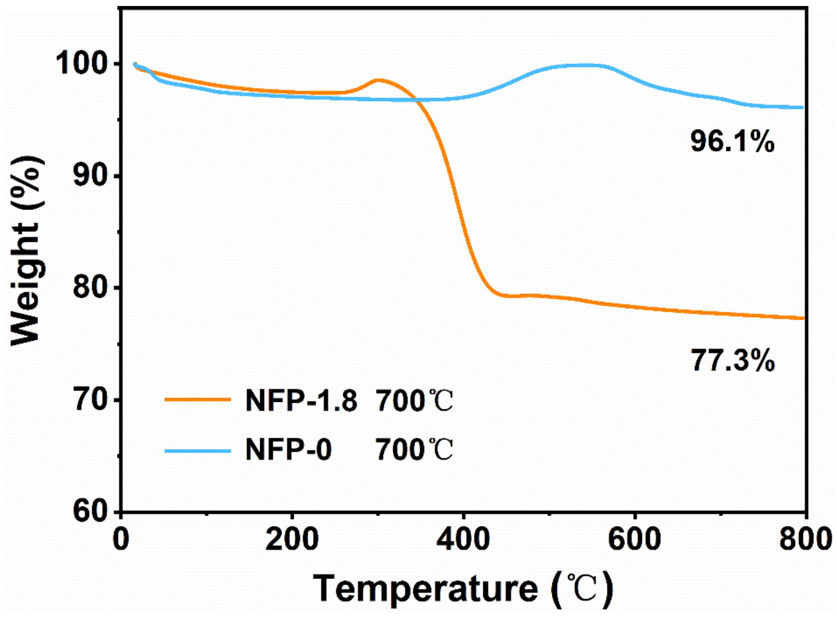

Figure S6. TG curves of NFP-1.8 and NFP-0.

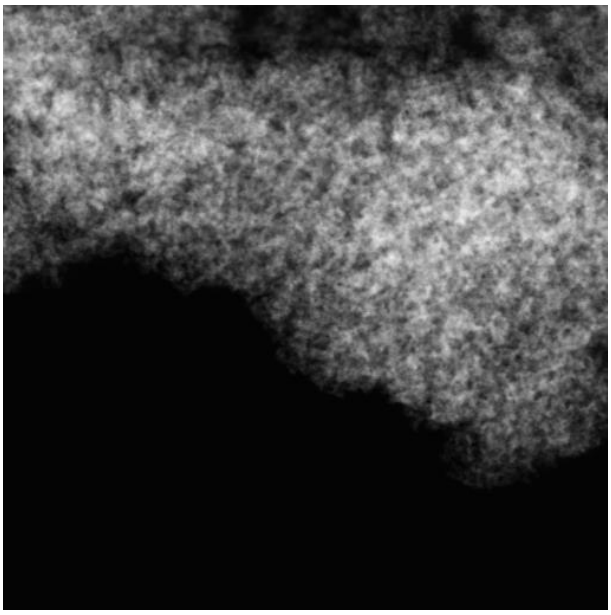

Figure S7. HAADF-STEM pattern of NFP-1.8.

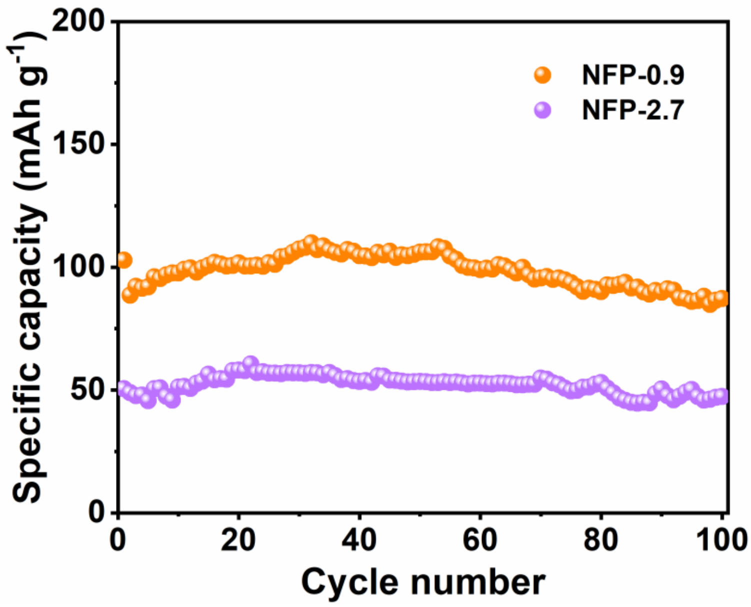

Figure S8. Cycling stability of NFP-0.9 and NFP-2.7 at a current density of 20 mA g^{-1}.

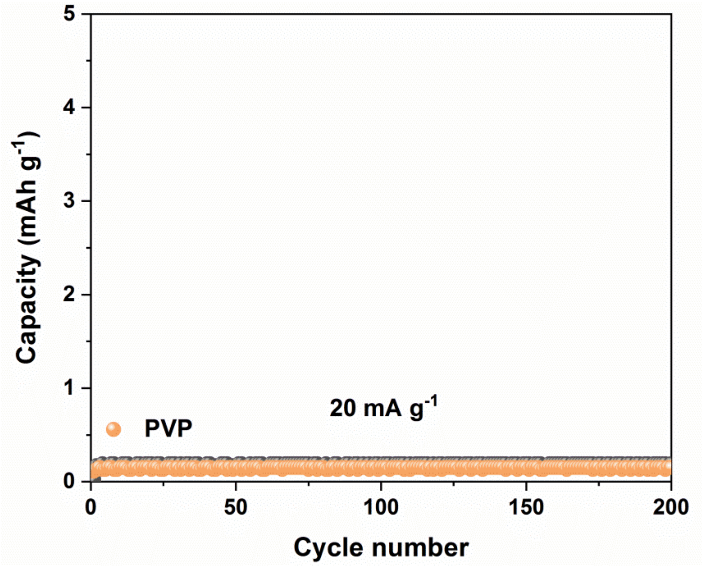

Figure S9. The electrochemical performance of PVP electrode at 20 mA g^{-1}.

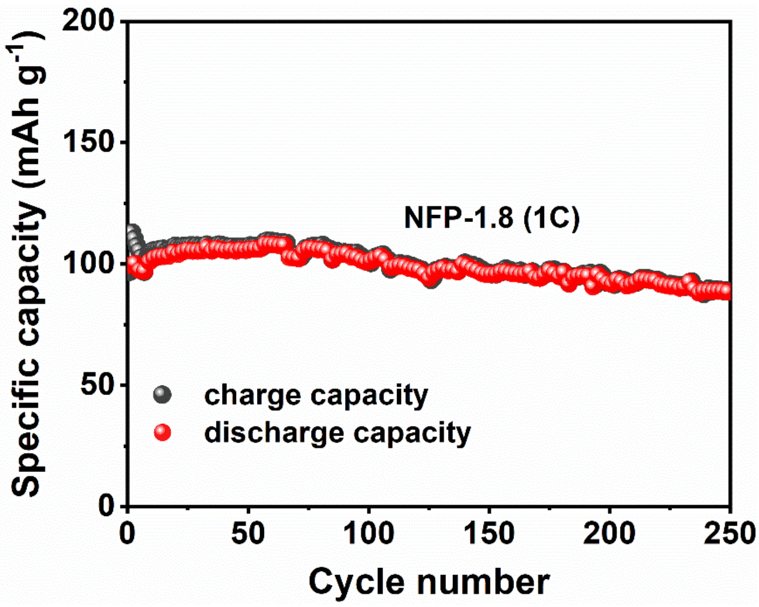

Figure S10. Cycling stability of NFP-1.8 at 1 C.

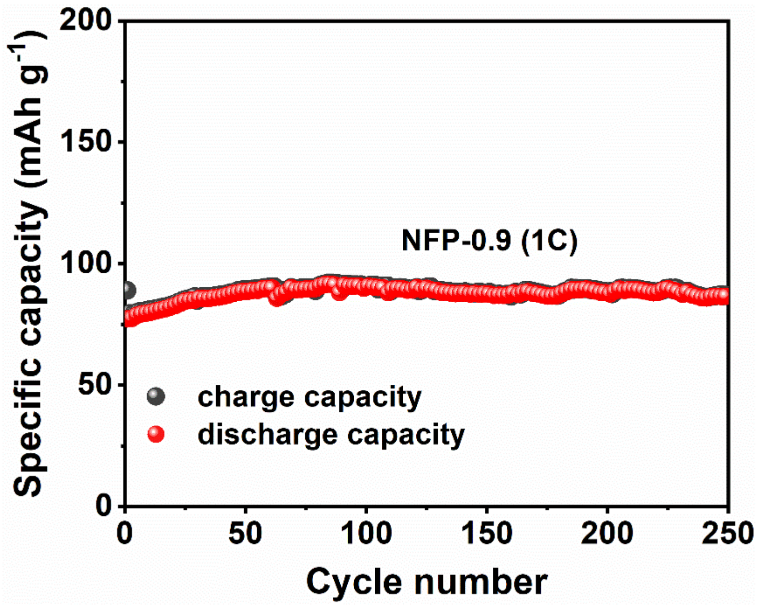

Figure S11. Cycling stability of NFP-0.9 at 1 C.

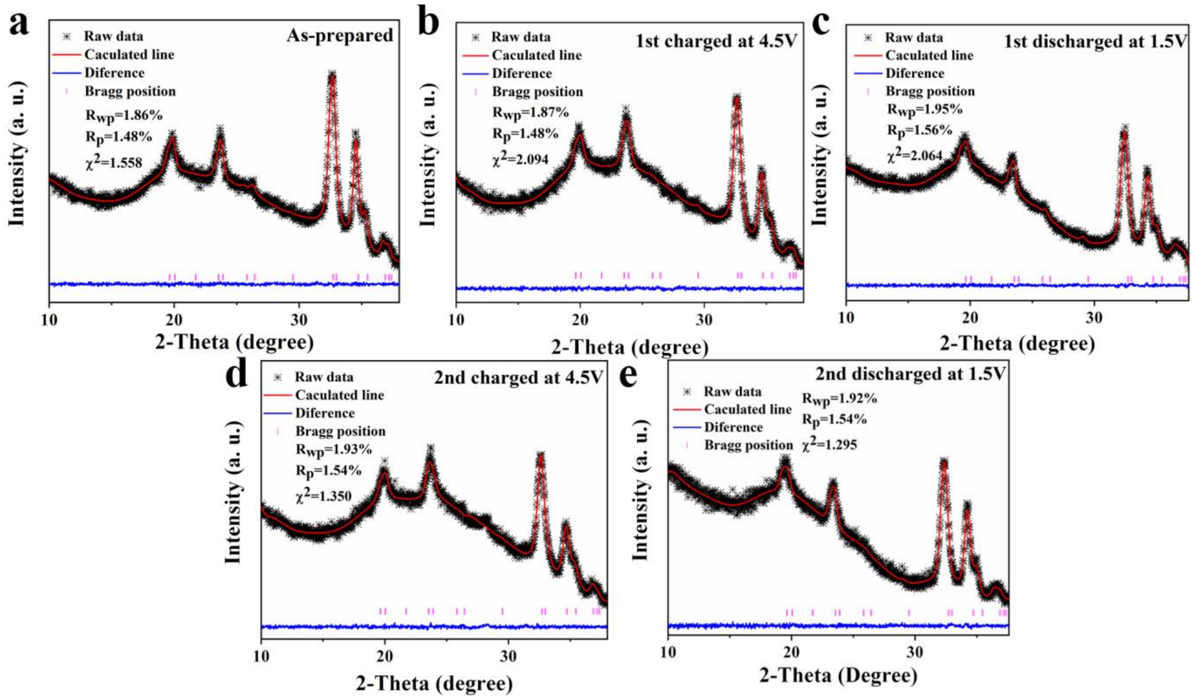

Figure S12. The Rietveld refinement X-ray diffraction patterns of NFR-1.8 cathode at different charge/discharge states.

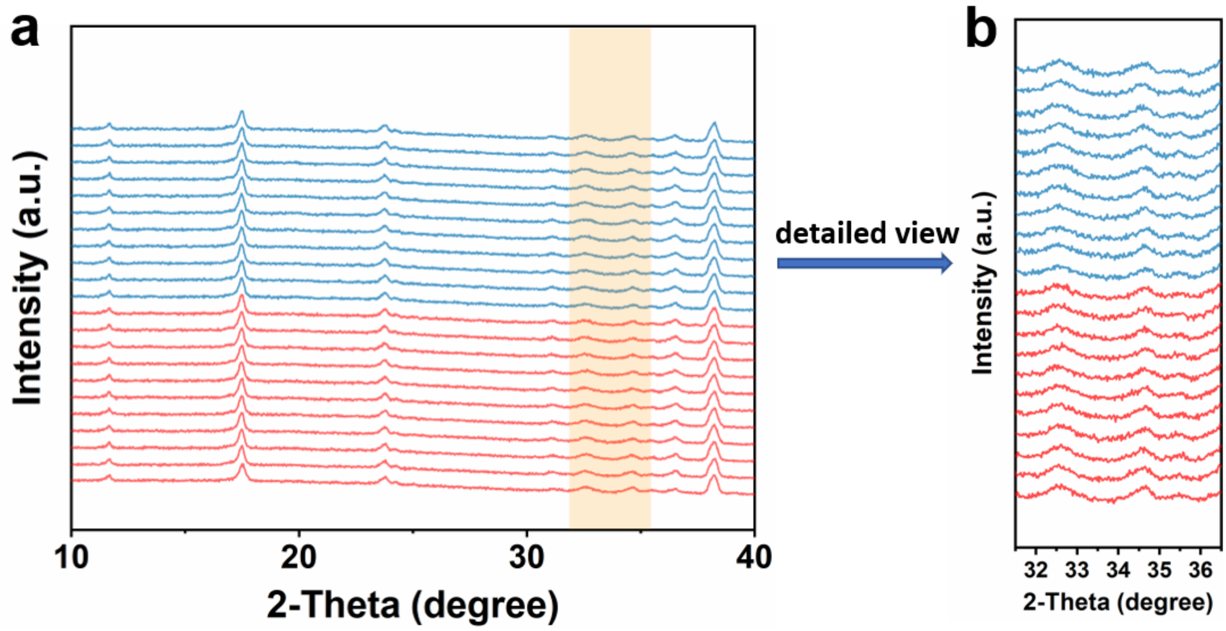

Figure S13. (a) In situ XRD patterns of NFP-1.8 electrode collected during the first charge-discharge process under a current rate of 0.2 C; (b) the detailed view.

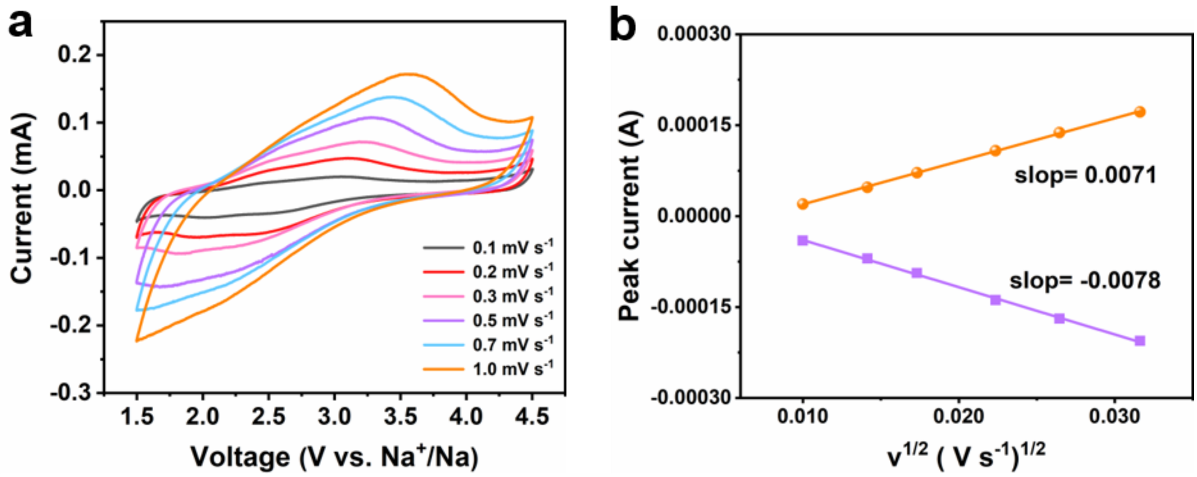

Figure S14. (a) CV curves at various scan rates from 0.1 to 1.0 mV s^{-1} and (b) the corresponding linear relationship between the peak currents and the square root of scan rates ($v^{1/2}$) of NFP-1.8 cathode.

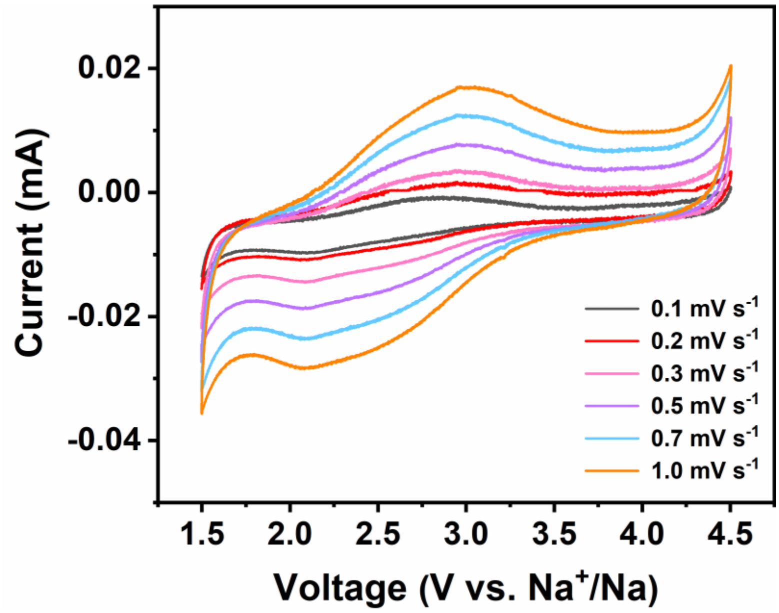

Figure S15. CV curves at various scan rates from 0.1 to 1.0 mV s^{-1} of NFP-0 cathode.

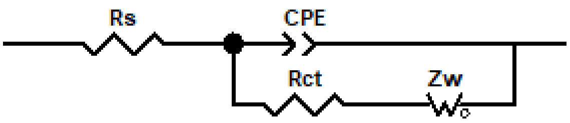

Figure S16. An equivalent circuit was used for fitting the experimental data.

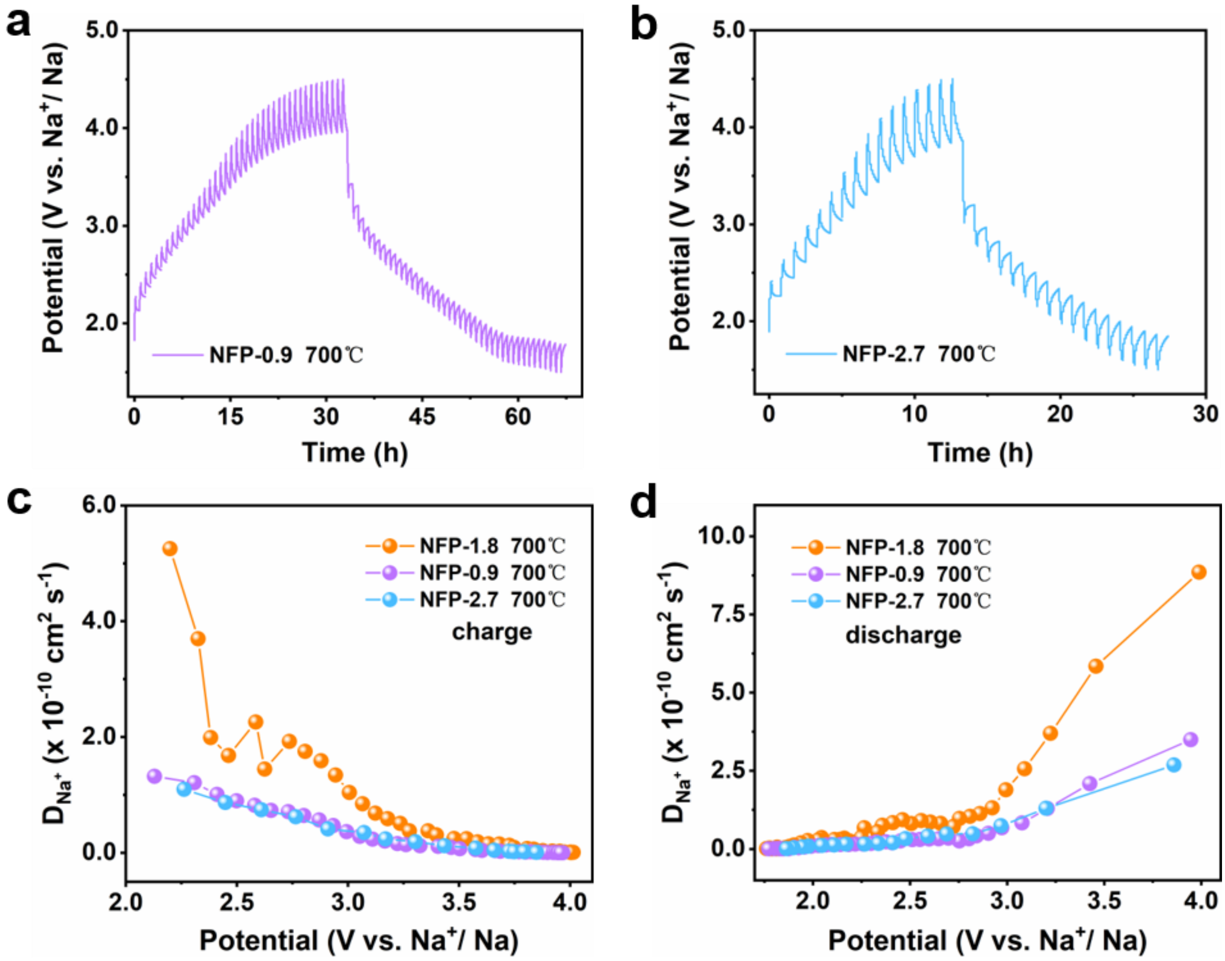

Figure S17. GITT curves of (a) NFP-0.9 and (b) NFP-2.7 electrodes; Comparison of Na^+ diffusion coefficients of (c) charge and (d) discharge process.

Table S1. The comparison of sodium-ion storage performance between NFP-1.8 and the previously reported results.

Material	Cycling performance	Retention (%)	Year/Reference
maricite $NaFePO_4$@C	110 mA h g^{-1} at 0.1C after 50 cycles	80%	2019/[1]
maricite $NaFePO_4$	40 mA h g^{-1} at 0.05C in the 1st cycle at 298 K	-	2017/[2]
	100 mA h g^{-1} at 0.5C after 120 cycles at 363 K	-	
maricite $NaFePO_4$/C	48.8 mA h g^{-1} at 0.05C after 50 cycles	100%	2017/[3]
olivine $NaFePO_4$ in high salt concentration ionic liquid electrolytes	80 mA h g^{-1} at 0.5C after 100 cycles at 50 °C	95%	2018/[4]
ball-milled maricite $NaFePO_4$	47 mA h g^{-1} at 1C after 100 cycles	67.2%	2018/[5]
triphylite-$NaFePO_4$	101 mA h g^{-1} at 0.1C after 200 cycles	76.5%	2020/[6]
$NaFePO_4$ nanoparticles	28 mA h g^{-1} at 0.1C after 35 cycles	92%	2021/[7]
olivine $NaFePO_4$/C microsphere	100 mA h g^{-1} at 0.1C after 240 cycles	90%	2015/[8]
maricite $NaFePO_4$	63 mA h g^{-1} at 0.05C after 150 cycles	-	2014/[9]
$Na_2Fe_2(SO_4)_3$@C@GO	107.9 mA h g^{-1} at 0.1C 300 cycles at 0.2C	90.1%	2018/[10]
$Na_2Fe_2(SO_4)_3$	64 mA h g^{-1} at 0.05C after 50 cycles	82%	2021/[11]
$Na_2Fe(SO_4)_2$	82 mA h g^{-1} at 0.1C 100 cycles at 0.5C	84%	2019/[12]
$Na_4Fe_3(PO_4)_2(P_2O_7)$/C	94 mA h g^{-1} at 0.5C after 300 cycles	90.8%	2021/[13]

$Na_4Fe_3(P_2O_7)(PO_4)$	90 mA h g^{-1} at 1C after 200 cycles	87.4%	2022/[14]
$Na_4Fe_3(PO_4)_2(P_2O_7)$@MCNTs	115.7 mA h g^{-1} at 0.1C 1200 cycles at 2C	95%	2020/[15]
**maricite NFP-1.8**	**101.4 mA h g^{-1} at 20 mA g^{-1} after 100 cycles**	**90.5%**	**This work**

Table S2. The volume change of the NFR-1.8 cathode for the first two cycles.

Sample	As prepared	1st charged at 4.5 V	1st discharged at 1.5 V	2nd charged at 4.5 V	2nd discharged at 1.5 V
volume change (%)	0	0.70	0.07	0.81	0.20

References

[1] D. Wang, Y. B. Wu, J. M. Lv, R. R. Wang, S. L. Xu, *Colloids Surf. A* **2019**, *583*, 123957.

[2] J. Hwang, K. Matsumoto, T. Nohira, R. Hagiwara, *Electrochemistry* **2017**, *85*, 675.

[3] L. Zhao, D. M. Zhou, W. X. Huang, X. Y. Kang, Q. W. Shi, Z. L. Deng, X. W. Yan, Y. B. Yu, *Int. J. Electrochem. Sci.* **2017**, *12*, 3153.

[4] M. Hilder, P. C. Howlett, D. Saurel, H. Anne, M. Casas-Cabanas, M. Armand, T. Rojo, D. R. MacFarlane, M. Forsyth, *J. Power Sources* **2018**, *406*, 70.

[5] J. Hwang, K. Matsumoto, Y. Orikasa, M. Katayama, Y. Inada, T. Nohira, R. Hagiwara, *J. Power Sources* **2018**, *377*, 80.

[6] C. Berlanga, I. Monterrubio, M. Armand, T. Rojo, M. Galceran, M. Casas-Cabanas, *ACS Sustainable Chem. Eng.* **2020**, *8*, 725.

[7] S. N. Yadav, S. J. Rajoba, R. S. Kalubarme, V. G. Parale, L. D. Jadhav, *Chinese J. Phys.* **2021**, *69*, 134.

[8] Y. J. Fang, Q. Liu, L. F. Xiao, X. P. Ai, H. X. Yang, Y. L. Cao, *ACS Appl. Mater. Interfaces* **2015**, *7*, 17977.

[9] P. P. Prosini, C. Cento, A. Masci, M. Carewska, *Solid State Ionics* **2014**, *263*, 1.

[1] D. Wang, Y. B. Wu, J. M. Lv, R. R. Wang, S. L. Xu, *Colloids Surf. A* **2019**, *583*, 123957.

[2] J. Hwang, K. Matsumoto, T. Nohira, R. Hagiwara, *Electrochemistry* **2017**, *85*, 675.

[3] L. Zhao, D. M. Zhou, W. X. Huang, X. Y. Kang, Q. W. Shi, Z. L. Deng, X. W. Yan, Y. B. Yu, *Int. J. Electrochem. Sci.* **2017**, *12*, 3153.

[4] M. Hilder, P. C. Howlett, D. Saurel, H. Anne, M. Casas-Cabanas, M. Armand, T. Rojo, D. R. MacFarlane, M. Forsyth, *J. Power Sources* **2018**, *406*, 70.

[5] J. Hwang, K. Matsumoto, Y. Orikasa, M. Katayama, Y. Inada, T. Nohira, R. Hagiwara, *J.*

Power Sources **2018**, *377*, 80.

[6] C. Berlanga, I. Monterrubio, M. Armand, T. Rojo, M. Galceran, M. Casas-Cabanas, *ACS Sustainable Chem. Eng.* **2020**, *8*, 725.

[7] S. N. Yadav, S. J. Rajoba, R. S. Kalubarme, V. G. Parale, L. D. Jadhav, *Chinese J. Phys.* **2021**, *69*, 134.

[8] Y. J. Fang, Q. Liu, L. F. Xiao, X. P. Ai, H. X. Yang, Y. L. Cao, *ACS Appl. Mater. Interfaces* **2015**, *7*, 17977.

[9] P. P. Prosini, C. Cento, A. Masci, M. Carewska, *Solid State Ionics* **2014**, *263*, 1.

[10] M. Chen, D. Cortie, Z. Hu, H. Jin, S. Wang, Q. Gu, W. Hua, E. Wang, W. Lai, L. Chen, S. L. Chou, X. L. Wang, S. X. Dou, *Adv. Energy Mater.*, **2018**, 8, 1800944.

[11] B. Nawaz, M. O. Ullah. *J. Mater. Sci-Mater. El.* **2021**,32, 14509.

[12] W. Pan, W. Guan, S. Liu, B. B. Xu, C. Liang, H. Pan, M. Yan, Y. Jiang. *J. Mater. Chem. A* **2019**,7, 13197.

[13] L.-m. Zhang, X.-d. He, S. Wang, N.-q. Ren, J.-r. Wang, J.-m. Dong, F. Chen, Y.-x. Li, Z.-y. Wen, C.-h. Chen. *ACS Appl. Mater. Interfaces* **2021**,13, 25972.

[14] X. Li, Y. Meng, D. Xiao. *Chem. Eur. J.* **2023**,29. 12: e202203381.

[15] Y. Cao, X. Xia, Y. Liu, N. Wang, J. Zhang, D. Zhao, Y. Xia. J. Power Sources **2020**,461, 228130.
